# User of angiotensin-converting-enzyme inhibitor and/or angiotensin II receptor blocker might be associated with vascular calcification in predialysis chronic kidney disease patients: a retrospective single-center observational study: ACEI/ARB and vascular calcification

**DOI:** 10.1186/s12882-020-02198-6

**Published:** 2021-01-06

**Authors:** Kaori Takaori, Hirotsugu Iwatani, Masafumi Yamato, Takahito Ito

**Affiliations:** 1 Department of Nephrology, National Hospital Organization Osaka National Hospital, 2-1-14, Hoenzaka, Chuoku, Osaka, 540-0006 Japan; 2 Present Affiliation: Department of Nephrology, Yodogawa Christian Hospital, 1-7-50, Kunijima, Higashi Yodogawa Ku, Osaka, 533-0024 Japan; 3 Kataguilli Medical Center, 4-3-9 Sumiyoshi-cho, Shibata, Niigata, 957-0061 Japan

**Keywords:** ACE inhibitor, ARB, Angiotensin II, Vascular calcification

## Abstract

**Background:**

Vascular calcification is a prominent feature in chronic kidney disease (CKD) and diabetes mellitus. A recent report suggests that angiotensin II is protective to vascular calcification. Therefore, we investigated the relationship between vascular calcification and use of angiotensin-converting-enzyme inhibitor (ACEI) and/or angiotensin II receptor blocker (ARB) from a cross-sectional view.

**Methods:**

A total of 121 predialysis CKD patients (age 71 ± 12 y; male 72; estimated glomerular filtration rate (eGFR) 20.2 (11.8 - 40.3) mL/min/1.73 m^2^) who underwent thoracoabdominal plain computed tomography scan were included in this study. The total vascular calcification volume (Calc) was calculated with a three-dimensional imaging software and standardized by body surface area (BSA). The relevance between log [Calc/BSA] and ACEI/ARB use was investigated by multivariate linear regression analyses with or without a time-duration factor of ACEI/ARB use.

**Results:**

The Calc/BSA was 5.62 (2.01 - 12.7) mL/m^2^ in 121 patients. In multivariate analyses adjusted with age, sex, ACEI/ARB and log [eGFR], ACEI/ARB use is significantly and positively associated with log [Calc/BSA] (β = 0.2781, *p* = 0.0007). Even after the adjustment by age, sex, log [eGFR], phosphate, diabetes mellitus, systolic blood pressure, warfarin, hypertension, dyslipidemia, low-density lipoprotein cholesterol, diuretics and ACEI/ARB, ACEI/ARB use is significantly and positively associated with log [Calc/BSA] (β = 0.1677, *p* = 0.0487). When 90 patients whose time-duration of ACEI/ARB use was clear in medical records were studied, a multivariate analysis adjusted with age, sex, log [eGFR], and ACEI/ARB duration factors showed that the longer use of ACEI/ARB more than 2 years was significantly, independently and positively associated with log [Calc/BSA] (β = 0.2864, *p* = 0.0060).

**Conclusions:**

ACEI/ARB user was associated with vascular calcification in predialysis patients with low eGFR. Prospective studies with larger numbers of patients or more in vitro studies are needed to confirm whether this phenomenon is due to the use of ACEI/ARB itself, the underlying disease condition or the prescription bias.

**Supplementary Information:**

The online version contains supplementary material available at 10.1186/s12882-020-02198-6.

## Background

Vascular calcification is often found in patients with chronic kidney disease (CKD) or diabetes mellitus (DM), and is closely associated with cardiovascular events and mortality [1, 2]. Vascular calcification is formed by actively regulated processes between inductive and inhibitory factors, similar to those in bone formation and remodeling [3].

Anatomically, vascular calcification is classified into two types: intimal and medial calcification. The intimal layer of the vascular walls is mainly composed of endothelium and is commonly associated with atherosclerotic plaques in pathologic conditions. Therefore, intimal calcification often leads to stenosis or occlusion of the vessels, as in coronary artery disease. In contrast, the medial layer of the vascular walls is composed of smooth muscle cells and elastin-rich extracellular matrix. Medial calcification, also known as Mönckeberg’s calcification, is often associated with CKD. As medial calcification affects elastin-rich matrixes, a reduction or loss of vascular elasticity or vascular tone is the major problem, rather than stenosis of the vascular lumen. Many clinical studies suggest that hypertension, DM, dyslipidemia, aging, smoking, and genetic factors, which are known as “traditional” Framingham risk factors, promote vascular calcification. Non-traditional risk factors, including inflammation, oxidative stress, advanced glycation end products, and abnormal mineral metabolism (e.g. high serum phosphate) are also associated with vascular calcification [4, 5].

The renin-angiotensin system is known to be a major pathogenic factor in vascular smooth muscle cell apoptosis, growth, and differentiation. Suggestively, it is likely to play a role in calcification [6]. Calcified arteries show an upregulation of angiotensin 1 receptors, and treatment with an angiotensin II type 1 receptor blocker has been shown to inhibit calcification in a rabbit model [7]. Most recently, another study showed angiotensin II type 2 receptor (AT2)-mediated attenuation of vascular calcification, which was induced by an adenine and high-phosphate diet [8], suggesting that ARB might be protective to vascular calcification. In contrast, in 5/6 nephrectomized rats (a model of CKD), treatment with enalapril improved myocardial hypertrophy and progression of renal disease, but had no effect on vascular calcification [9].

But, a recent report indicated that angiotensin II prevents phosphate-induced calcification in human aortic smooth muscle cells in vitro by increasing magnesium influx, activating ERK1/2, and inhibiting canonical Wnt/beta-catenin signaling pathways [10]. This study mimicked medial calcification in patients with reduced renal function and high phosphate levels. Therefore, we investigated the relationship between vascular calcification and use of angiotensin-converting-enzyme inhibitor (ACEI) and/or angiotensin II receptor blocker (ARB).

## Methods

### Study population

We retrospectively investigated 152 patients who underwent thoracoabdominal plain computed tomography (CT) scan between May 2014 and April 2018 in the department of nephrology, National Hospital Organization Osaka National Hospital. We excluded 31 individuals because of maintenance dialysis (14 cases), continuous hemodiafiltration (2 cases), fulminant hepatitis (1 case), a history of aortic aneurysm treatment or surgical operation to major large arteries (8 cases), extremely low value of creatinine due to the decreased muscle volume (1 case), lacking data of blood pressure on the day of CT scan (1 case), and/or lacking lipid data profile within 15 days from the day of CT scan (5 cases). One individual had two conditions of the above-mentioned exclusion criteria. In the remaining 121 patients, clinical data, including laboratory tests, past history, comorbidities, and medications, were collected. DM was defined as a casual blood glucose level ≧200 mg/dL, hemoglobin A1c≧6.5%, or the use of antidiabetic medications. Hypertension was defined as systolic blood pressure ≧140 mmHg, diastolic blood pressure ≧90 mmHg, or use of antihypertensives. Dyslipidemia was defined as a low-density lipoprotein cholesterol (LDL-C) level ≧140 mg/dL, high-density lipoprotein cholesterol level <40 mg/dL, triglyceride level ≧150 mg/dL, or the use of lipid-lowering medications. Systolic blood pressure (SBP) on the day of CT scan was collected. Current prescription of warfarin, ACEI and/or ARB on the day of CT scan was collected from medical records. ACEI and/or ARB were basically prescribed to patients with CKD such as those accompanying decreased eGFR or proteinuria, patients with cardiovascular diseases or patients with DM especially with proteinuria/albuminuria.

### Laboratory analyses

Basically, the results of blood tests performed within 3 days of the thoracoabdominal plain CT scan were collected. LDL-C was calculated by Friedewald formula (LDL-C = Total Cholesterol – high-density lipoprotein cholesterol – Triglyceride/5) [11]. These lipid data were used within 15 days of the thoracoabdominal plain CT scan. In case where the calculation was impossible, LDL-C was collected from the directly measured values. In cases with a serum albumin level < 4.0 g/dL, the serum calcium level (mg/dL) was adjusted with Payne’s formula as follows: adjusted serum calcium level (mg/dL) = serum calcium level (mg/dL) + (4.0 – albumin (g/dL)). The estimated glomerular filtration rate (eGFR) was calculated using the following equation, which was defined by the Japanese Society of Nephrology: eGFR = 194 × serum creatinine level (mg/dL) ^-1.094^ × age (years) ^-0.287^ (× 0.739 if female) (mL/min/1.73m^2^). Body surface area (BSA) for our Japanese patients was calculated with Fujimoto’s formula as follows: BSA = body weight (kg) ^0.444^ x height (cm) ^0.663^ × 0.008883 [12].

### CT protocol and image analysis

All CT examinations were performed using a commercially available CT system (Discovery CT750 HD, GE Healthcare, Japan; SOMATOM Definition AS+, Siemens, Erlangen, Germany). The CT protocol comprised the following parameters: tube voltage, 120 kV; tube current, modulated with automated exposure control; and gantry rotation time, 500 ms. To reconstruct the raw image data, we used a section thickness of 1 mm, field of view of approximately 360 mm, and a matrix of 512 × 512. The vascular calcification volume (Calc) was measured using a 3-D imaging software (Volume analyzer SYNAPSE VINCENT, FUJIFILM Medical Co., Ltd., Tokyo). The method was similar to that in Kinugasa et al. [13]. First, we reconstructed a 3-D image from the original CT image. Second, bone was removed by the software. Third, the total aorta (from the apex level of the lung to the femoral artery) was extracted. Using a Hounsfield unit of more than 130 as the cut-off level, we obtained the total vascular calcification volume semi-automatically by the software.

### The relationship between log [Calc/BSA] and clinical parameters including use of warfarin, ACEI and/or ARB

The relationship between log [Calc/BSA] and either of age, log [eGFR], SBP, use of warfarin, use of diuretics, presence of malignancy, or use of ACEI and/or ARB was investigated by univariate linear regression analyses as shown in Fig. 2 and Fig. 3. Then, log [Calc/BSA] was adjusted by clinical parameters in multivariate linear regression analyses as shown in each model in Table 2.

### The relationship between log [Calc/BSA] and the duration of ACEI/ARB

To further investigate the accumulative effect of ACEI/ARB on vascular calcification, we added the duration of ACEI/ARB as an explanatory factor. Because the subject number was small, we classified the subject into 3 groups based on the duration of ACEI/ARB until the day of CT performed: Group 1 (none or less than 6 months), Group 2 (between 6 months and 2 years) and Group 3 (more than 2 years). Because 31 patients were unknown as to the duration of ACEI/ARB, we excluded these 31 patients in the following analyses. In the remaining 90 patients, log [Calc/BSA] was compared among group 1, group 2 and group 3 by Tukey-Kramer HSD test. The relevance between log [Calc/BSA] and the duration of ACEI/ARB was investigated by tendency analysis (Jonckheere-Terpstra trend test) and multivariate linear regression analysis.

### Statistical analysis

Data are reported as percentages or means ± standard deviation (SD) for normally distributed variables, and as medians (interquartile range) for non-normally distributed variables. To minimize skewness, the eGFR and Calc/BSA were transformed using the natural logarithm. Calc/BSA was confirmed not to include 0 value in our 121 patients. The relevance between log [Calc/BSA] and ACEI/ARB use was investigated by several multivariate linear regression analyses. Statistical analyses were performed using JMP (ver14, SAS Institute Japan, Tokyo, Japan) and Excel add-in software (Social Survey Research Information Co., Ltd. Tokyo, Japan). In the multivariate analysis, *p* < 0.05 was considered statistically significant.

## Results

**Fig. 1:**
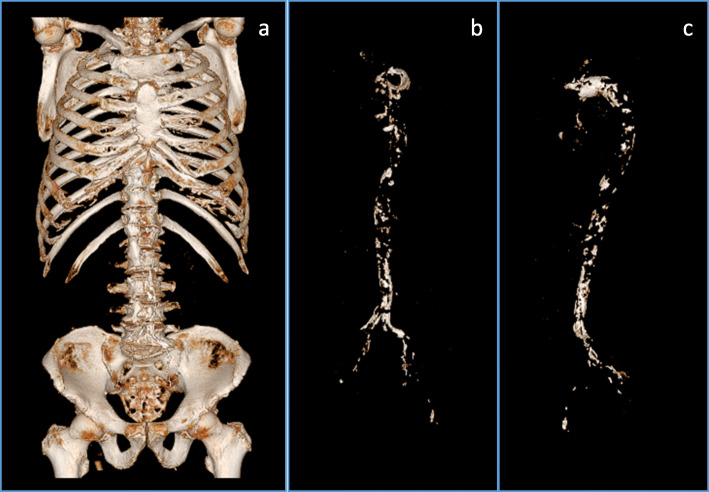
Representative image of vascular calcification using 3D imaging software. 3D images from an original CT image were reconstructed (**a**). After the removal of bone and the manual confirmation, the vascular calcification image is shown in (**b**). Rotation of the vascular calcification image (**b**) is shown in (**c**)

Our study subjects underwent CT scan in order to investigate the presence or absence of malignancy (91 cases), locate the source of infection (18 cases), evaluate aortic aneurysm or aortic dissection (4 cases), investigate the cause of anemia (4 cases), evaluate renal artery calcification (2 cases), and for other miscellaneous reasons (9 cases). A single CT examination often detected more than one such condition in a total of 121 patients. A representative vascular calcification image is shown in Fig. 1.

**Fig. 2:**
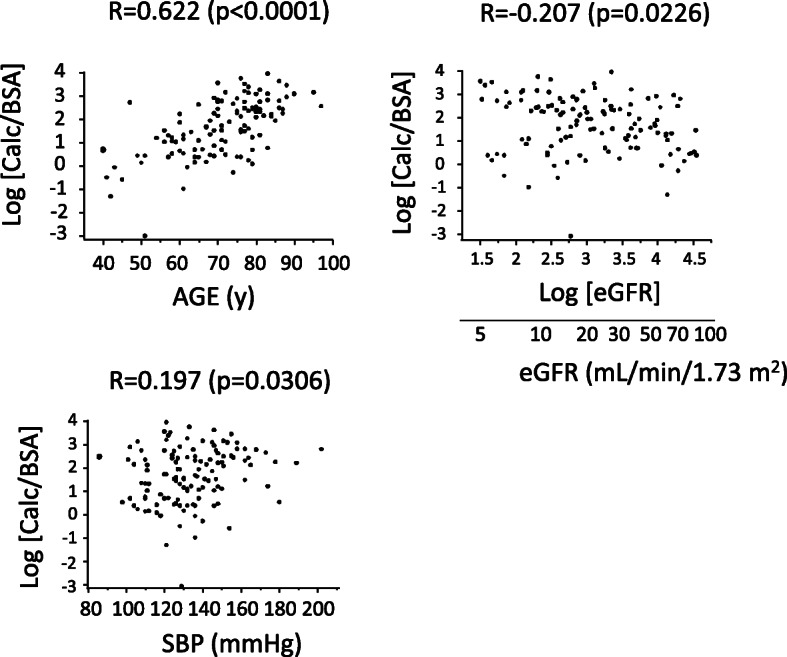
Univariate analyses of age, eGFR, and systolic blood pressure (SBP) on the day of CT scan against vascular calcification volume. In univariate analyses, phosphate concentration and malignancy were not associated with log [Calc/BSA] (*p* = 0.3768 and *p* = 0.8414, respectively). Male sex, diabetes and diuretics were significantly associated with log [Calc/BSA] (*p* = 0.0002, *p* = 0.0013 and *p* = 0.0020, respectively). BSA, body surface area calculated with Fujimoto’s formula; Calc, calcification volume; CT, computed tomography; eGFR, estimated glomerular filtration rate; SBP, systolic blood pressure

**Fig. 3:**
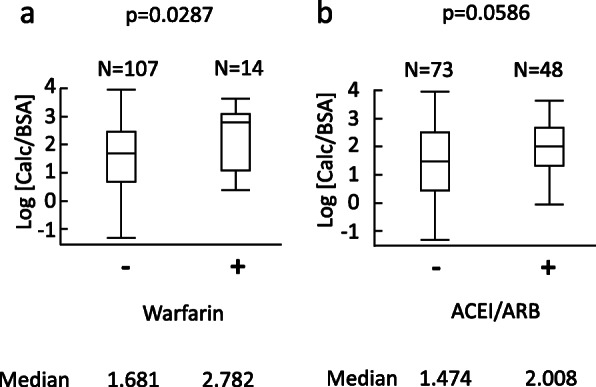
The relationship between warfarin or ACEI/ARB and vascular calcification volume. In univariate analyses, use of warfarin was significantly associated with vascular calcification volume (*p* = 0.0287). ACEI/ARB was marginally associated with vascular calcification volume (*p* = 0.0586). **a**, warfarin. **b**, ACEI/ARB. ACEI, angiotensin-converting-enzyme inhibitor; ARB, angiotensin II receptor blocker; BSA, body surface area calculated with Fujimoto’s formula; Calc, calcification volume

The characteristics of the 121 patients (mean age, 71 ± 12 years; 72 men) included in the analysis are provided in Table 1A. Among these patients, the median Calc was 8.94 (3.26 - 18.9) mL, and the median Calc/BSA was 5.62 (2.01 - 12.7) mL/m^2^. The mean log [Calc/BSA] was 1.67 ± 1.20. The mean log [eGFR] was 3.1 ± 0.8, which corresponds to an eGFR of 20.2 (11.8 - 40.3) mL/min/1.73m^2^. The relationships between log [Calc/BSA] and either of age, log [eGFR], SBP, warfarin, diuretics, malignancy or ACEI/ARB were investigated. Log [Calc/BSA] was significantly associated with age (*p* < 0.0001), log [eGFR] (*p* = 0.0226) or systolic blood pressure (*p* = 0.0306) as shown in Fig. 2. The association of warfarin with log [Calc/BSA] was significant (*p* = 0.0287). The association of ACEI/ARB with log [Calc/BSA] was marginally significant (*p* = 0.0586) (Fig. 3) Moreover, the association of diuretics with log [Calc/BSA] was significant (*p* = 0.0020). The association of malignancy with log [Calc/BSA] was not significant (*p* = 0.8414). In univariate analyses, the difference of the median values of log [Calc/BSA] was 1.10 with or without warfarin (Fig. 3). That was 0.53 with or without ACEI/ARB (Fig. 3).

**Table 1:** Patients characteristics

A		B	
N	121	N	90
Age, years	71 ± 12	Age, years	72 (63–78)
Men, n (%)	72 (60)	Men, n (%)	54 (60)
Serum phosphate (mg/dL)	3.9 ± 1.1	Serum phosphate (mg/dL)	3.9 ± 1.1
eGFR (mL/min/1.73 m2)eGFR (mL/min/1.73 m2)	20.2 (11.8–40.3)	eGFR (mL/min/1.73 m2)eGFR (mL/min/1.73 m2)	21.3 (12–50.2)
Log [eGFR]	3.1 ± 0.8	Log [eGFR]	3.1 ± 0.8
Calcification Volume (mL)	8.94 (3.26–18.9)	Calcification Volume (mL)	8.0 (3.1–19.3)
Calc/BSA (mL/m2)Calc/BSA (mL/m2)	5.62 (2.01–12.7)	Calc/BSA (mL/m2)Calc/BSA (mL/m2)	5.26 (1.96–13.2)
Log [Calc/BSA]	1.67 ± 1.20	Log [Calc/BSA]	1.66 (0.67–2.58)
Systolic Blood Pressure (mmHg)	134 ± 20	Systolic Blood Pressure (mmHg)	133 ± 20
Hypertension (n(%))	98 (81)	Hypertension (n(%))	69 (77)
Diabetes mellitus (n (%))	54 (45)	Diabetes mellitus (n (%))	38 (42)
Dyslipidemia (n (%))	69 (57)	Dyslipidemia (n (%))	49 (54)
LDL-C (mg/dL)	118 ± 66	LDL-C (mg/dL)	116 ± 65
Malignancy (n (%))	35 (29)	Malignancy (n (%))	30 (33)
Medications		Medications	
Warfarin User (n (%))	14 (12)	Warfarin User (n (%))	12 (13)
Vitamin D User (n (%))	12 (10)	Vitamin D User (n (%))	8 (9)
Phosphate-Binding Agent User (n (%))	13 (11)	Phosphate-Binding Agent User (n (%))	8 (9)
Diuretics user (n (%))	67 (55)	Diuretics user (n (%))	50 (56)
ACEI/ARB User (n (%))	48 (40)	ACEI/ARB Duration	
		None or < 6 months (n (%))	42 (47)
		6 months ≤ < 2 years (n (%))	7 (8)
		2 years ≤ (n (%))	41 (46)

(A) *N* = 121, (B) *N* = 90 where patients whose history of ACEI/ARB use were unknown are excluded. *eGFR* estimated glomerular filtration rate, *Calc* calcification volume, *BSA* body surface area calculated with Fujimoto’s formula, *LDL-C* low-density lipoprotein cholesterol, *ACEI* angiotensin-converting-enzyme inhibitor, *ARB* angiotensin II receptor blocker

Next, we performed multivariate linear regression analyses for the outcome of log [Calc/BSA] (Table 2). In model 3, where log [Calc/BSA] was adjusted by age, sex log [eGFR] and ACEI/ARB use, ACEI/ARB use showed a positive and significant association with log [Calc/BSA]. When the explanatory variables such as phosphate, DM or SBP were further added as in model 5 or model 6, ACEI/ARB use still showed a positive and significant association (Table 2). When the explanatory variables such as presence of hypertension, presence of hyperlipidemia, LDL-C, use of diuretics, or presence of malignancy were further added as in model 7, model 8 or model 9, ACEI/ARB use still showed a positive and significant association with log [Calc/BSA] (Table 2). The range of β value of ACEI/ARB in various multivariate models is from 0.1671 to 0.2781, which indicates that the differences of log [Calc/BSA] with or without ACEI/ARB were from 0.1671 to 0.2781 (Table 2). On the other hand, warfarin was not significant.

**Table 2:** Multivariate linear regression analyses for the outcome of log[Calc/BSA]

	Model 1	Model 2	Model 3	Model 4	Model 5	Model 6	Model 7	Model 8	Model 9
Adjusted R^2^	0.451	0.531	0.498	0.450	0.554	0.558	0.569	0.576	0.576
Age	0.0587 *p* < 0.0001	0.0639 *p* < 0.0001	0.0574 *p* < 0.0001	0.0577 *p* < 0.0001	0.0626 *p* < 0.0001	0.0617 *p* < 0.0001	0.0576 *p* < 0.0001	0.0607 *p* < 0.0001	0.0599 *p* < 0.0001
Male	0.2741 *p* = 0.0014	0.2474 *p* = 0.0025	0.3097 *p* = 0.0002	0.2695 *p* = 0.0017	0.2818 *p* = 0.0005	0.2859 *p* = 0.0004	0.2427 *p* = 0.0030	0.2513 *p* = 0.0020	0.2508 *p* = 0.0021
Log[eGFR]	−0.2344 *p* = 0.0214	−0.0603 *p* = 0.5993	−0.2573 *p* = 0.0086	−0.2268 *p* = 0.0264	−0.0872 *p* = 0.4386	− 0.0796 *p* = 0.4777	− 0.00033 *p* = 0.9978	− 0.00135 *p* = 0.9911	0.0100 *p* = 0.9338
Phosphate	–	0.2304 *p* = 0.0085	–	–	0.2161 *p* = 0.0116	0.2088 *p* = 0.0143	0.1707 *p* = 0.055	0.1503 *p* = 0.0874	0.1597 *p* = 0.0710
DM	–	0.2883 *p* = 0.0003	–	–	0.2373 *p* = 0.0027	0.2248 *p* = 0.0045	0.1892 *p* = 0.0174	0.1884 *p* = 0.0169	0.1819 *p* = 0.0213
SBP	–	–	–	–	–	0.0055 *p* = 0.1362	0.00552 *p* = 0.155	0.0056 *p* = 0.1497	0.00574 *p* = 0.1374
warfarin	–	–	–	0.1220 *p* = 0.3450	–	–	0.1295 *p* = 0.2664	–	0.1183 *p* = 0.3068
ACEI/ARB	–	–	0.2781 *p* = 0.0007	–	0.2028 *p* = 0.0107	0.1987 *p* = 0.0119	0.1677 *p* = 0.0487	0.1671 *p* = 0.0478	0.1685 *p* = 0.0461
Hypertension	–	–	–	–	–	–	0.1340 *p* = 0.2259	0.1130 *p* = 0.3048	0.1170 *p* = 0.2884
Dyslipidemia	–	–	–	–	–	–	−0.0808 *p* = 0.3340	−0.1013 *p* = 0.2258	− 0.0977 *p* = 0.2429
LDL-C	–	–	–	–	–	–	−0.00062 *p* = 0.6221	− 0.00073 *p* = 0.5620	− 0.00066 *p* = 0.6001
Diuretics	–	–	–	–	–	–	0.1696 *p* = 0.0392	0.1809 *p* = 0.0266	0.1745 *p* = 0.0327
Malignancy	–	–	–	–	–	–	–	−0.1412 *p* = 0.0929	− 0.1361 *p* = 0.1056

β values (upper line) and statistical *p* values (lower line) are shown in various regression models. The number of patients analyzed here are 121. The effects of age, phosphate, systolic blood pressure (SBP), and low-density lipoprotein cholesterol (LDL-C) are shown as per 1 year, per 1 mg/dL, per 1 mmHg and 1 mg/dL, respectively. *Calc*, calcification volume, *BSA* body surface area calculated with Fujimoto’s formula, *eGFR* estimated glomerular filtration rate, *DM* diabetes mellitus, *SBP* systolic blood pressure, *LDL-C* low-density lipoprotein cholesterol, *ACEI* angiotensin-converting-enzyme inhibitor, *ARB* angiotensin II receptor blocker

**Fig. 4:**
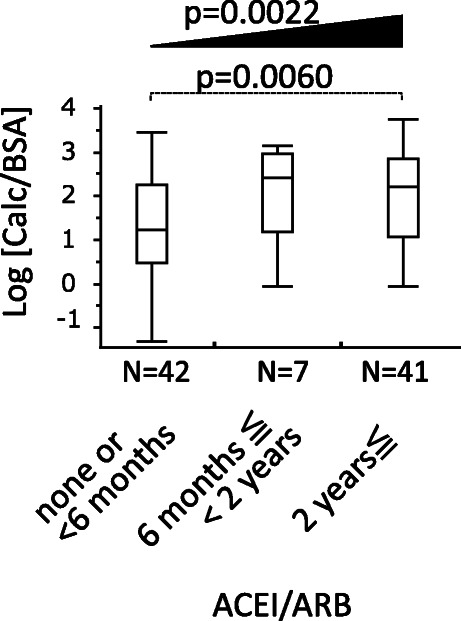
Time duration association of ACEI/ARB and vascular calcification volume. Patients using ACEI/ARB were categorized into three groups according to the duration time. Thirty-one patients were excluded from the analysis because of unknown duration time. Tukey-Kramer HSD test shows that the longest duration Group 3 has higher log [Calc/BSA] than the shortest Group 1 (*p* = 0.0060). Jonckheere-Terpstra trend test shows statistically significant increment of log [Calc/BSA] in accordance with the duration time (*p* = 0.0022). ACEI, angiotensin-converting-enzyme inhibitor; ARB, angiotensin II receptor blocker; BSA, body surface area calculated with Fujimoto’s formula; Calc, calcification volume

The duration of the use of ACEI/ARB is shown in Fig. 4. Because 31 patients were unknown as to the duration of ACEI/ARB, we excluded these 31 patients in the following analyses and the characteristics of the remaining 90 patients are shown in Table 1B. In 90 patients, group 3 had significantly higher log [Calc/BSA] than group 1 (*P* = 0.0060) with Tukey-Kramer HSD test. There was a significant trend that the longer the duration of ACEI/ARB, the higher log [Calc/BSA] (*P* = 0.0022) with Jonckheere-Terpstra trend test. In multivariate linear regression analysis where log [Calc/BSA] was adjusted by age, sex, log [eGFR], and the duration category of ACEI/ARB, the longer duration of ACEI/ARB more than 2 years showed an independent and positive association with log [Calc/BSA] (model A, Table 3). Even after the further adjustment by the various factors such as phosphate, DM, hypertension, dyslipidemia, diuretics or malignancy as in model B, model C and model D, ACEI/ARB treatment in Group 3 seems to consistently contribute to higher log [Calc/BSA] by 0.2268 to 0.2864 as compared with the reference group (Table 3).

**Table 3:** Time-duration association of ACEI/ARB use and vascular calcification volume

	Model A	Model B	Model C	Model D
Adjusted R^2^	0.493	0.559	0.587	0.597
Age	0.0575 *p* < 0.0001	0.0638 *p* < 0.0001	0.0573 *p* < 0.0001	0.0612 *p* < 0.0001
Male	0.2862 *p* = 0.0055	0.2946 *p* = 0.0031	0.2488 *p* = 0.0129	0.2587 *p* = 0.0092
Log [eGFR]	−0.1592 *p* = 0.1669	0.0202 *p* = 0.8804	0.1166 *p* = 0.4002	0.1099 *p* = 0.4222
Phosphate	–	0.2581 *p* = 0.0144	0.1928 *p* = 0.0649	0.1787 *p* = 0.0838
DM	–	0.2520 p = 0.011	0.2217 *p* = 0.0230	0.2037 *p* = 0.0351
Hypertension	–	–	0.2609 *p* = 0.0221	0.2517 *p* = 0.0254
Dyslipidemia	–	–	−0.1261 *p* = 0.2071	−0.1490 *p* = 0.1356
Diuretics	–	–	0.1836 *p* = 0.0602	0.1786 *p* = 0.0643
Malignancy	–	–	–	−0.1694 *p* = 0.0867
Group 1	reference	reference	reference	reference
Group 2	0.3186 *p* = 0.0854	0.1600 *p* = 0.3691	0.0825 *p* = 0.6372	0.0825 *p* = 0.6330
Group 3	0.2864 *p* = 0.0060	0.2605 *p* = 0.0124	0.2361 *p* = 0.0203	0.2268 *p* = 0.0241

Patients were categorized into three groups according to the duration time of ACEI/ARB. Thirty-one patients were excluded from the analysis because of unknown duration time. The effect of the time-duration of ACEI/ARB was analyzed by a multi-regression method using the group category as factors. Group 1, 2, and 3 denote as follows; Group 1 (none or less than 6 months), Group 2 (between 6 months and 2 years) and Group 3 (more than 2 years)

β values (upper line) and statistical p values (lower line) are shown in various regression models. The effects of age and phosphate are shown as per 1 year and per 1 mg/dL, respectively

*DM* diabetes mellitus, *ACEI* angiotensin-converting-enzyme inhibitor, *ARB* angiotensin II receptor blocker, *eGFR* estimated glomerular filtration rate

## Discussion

Our results suggest that ACEI/ARB user might be independently and significantly associated with vascular calcification in predialysis patients with low eGFR. However, the difference of log [Calc/BSA] with or without ACEI/ARB was about from 0.1671 to 0.2781 in multivariate analyses (Table 2). These correspond to 1.18–1.32 of Calc/BSA (mL/m^2^). Although the differences were statistically significant, the value was quite small. In other words, whether ACEI/ARB has a clinically significant impact on the vascular calcification or not remains unknown.

In the present study, use of warfarin was not associated with log [Calc/BSA] in multivariate analysis, but was significantly associated in the univariate analysis. Considering that warfarin inhibits matrix Gla protein, a vitamin K-dependent protein produced by vascular smooth muscle, and also a key inhibitor of vascular calcification [14], our results of the univariate analysis are almost compatible to the previously known fact. In the present study, use of diuretics was positively and significantly associated with log [Calc/BSA] in multivariate analysis as in model 7 or model 8 (Table 2). Considering that the use of diuretics induces hypomagnesemia [15] which is associated with vascular calcification [16], our results are compatible to the previously known fact. Even after the adjustment by the parameters including use of diuretics, use of ACEI/ARB was significantly and positively associated with log [Calc/BSA].

The present study mainly targeted the aorta in humans with reduced eGFR which is evident from representative image in Fig. 1, and we showed that ACEI/ARB user might be associated with vascular calcification. Our present results are consistent with a recent report by Herencia C. et al., showing the cause and effect relationship between angiotensin II inhibition and calcification in hyperphosphatemic milieu in vitro [10]. The in vitro study by Herencia C. et al. is based on the human aortic smooth muscle cells. Therefore, the in vitro study and our present study share a concept that the deterioration of human aortic calcification by blocking angiotensin II pathway both in clinical and in vitro point of view. In terms of vascular calcification and ACEI and/or ARB, Darabian S et al. reported that patients who received ACEI and/or ARB had a greater average of coronary artery calcification score [17], which is consistent with our results.

Vascular biology and bone biology have many regulatory mechanisms in common [3, 18]. In osteoblasts, angiotensin II significantly induces the expression of receptor activator of NF-kB ligand (RANKL), which leads to the activation of osteoclasts. These effects are completely cancelled by angiotensin II type 1 receptor blockers [19]. The renin-angiotensin system has a physiologic function in bone remodeling, and signaling via AT1a receptors negatively regulates bone turnover and bone mass [20, 21]. Therefore, we speculate that under reduced eGFR angiotensin II might prevent calcification in vessels, and that blockade of angiotensin II might promote vascular calcification.

The present study has several limitations. First, the study population comprised patients with CKD in the nephrology unit of Osaka National Hospital. Additionally, patients who undergo thoracoabdominal CT tend to have severe disorders. Therefore, a selection bias is unavoidable. Second, as this was a retrospective cross-sectional observational study, it cannot show causal relationships. The present study results do not completely rule out the possibility that patients on ACEI/ARB were formerly at high risk for vascular calcification. In other words, our present results might be the results of unobvious confounding factors. Thirdly, a prescription bias may be unavoidable. But we have already confirmed that there were no statistical differences among Group 1, Group 2 and Group 3 in terms of age, sex, albumin, corrected calcium, phosphate, product of corrected calcium and phosphate, log [eGFR], phosphate binder with calcium, phosphate binder without calcium, and SBP except for DM (Supplementary Data). Magnesium concentration was not included in Supplementary Data due to the substantial number of lacking data, however, there was no statistical difference between Group 1, Group 2 and Group 3. Lastly, the study population included patients with malignancy. However, the significant association of log [Calc/BSA] and ACEI/ARB was consistently maintained even after the adjustment by the presence of malignancy in multivariate analyses such as in model 8 (Table 2).

## Conclusions

ACEI/ARB user might be associated with vascular calcification in predialysis patients with low eGFR. This result is consistent with in vitro study showing that angiotensin II modulation influences calcification. Prospective studies with larger numbers of patients or more in vitro studies are needed to confirm whether the observed phenomenon is due to the use of ACEI/ARB itself, the underlying disease condition or the prescription bias.

## Supplementary Information

**Additional file 1.**

## Data Availability

The datasets used and/or analyzed during the current study are available from the corresponding author on reasonable request.

## Abbreviations

eGFR: Estimated glomerular filtration rate
Calc: Calcification volume
BSA: Body surface area calculated with Fujimoto’s formula
ACEI: Angiotensin-converting-enzyme inhibitor
ARB: Angiotensin II receptor blocker
CKD: Chronic kidney disease
DM: Diabetes mellitus
CT: Computed tomography
SBP: Systolic blood pressure
LDL-C: Low-density lipoprotein cholesterol

## References

[1] Moe SM, Chen NX (2008). Mechanisms of vascular calcification in chronic kidney disease. J Am Soc Nephrol.

[2] Iwatani H, Tomida K, Nagasawa Y, Imai E, Rakugi H, Isaka Y (2009). Massive and rapid left ventricular calcification. NDT Plus.

[3] Wu M, Rementer C, Giachelli CM (2013). Vascular calcification: an update on mechanisms and challenges in treatment. Calcif Tissue Int.

[4] Chen NX, Moe SM (2012). Vascular calcification: pathophysiology and risk factors. Curr Hypertens Rep.

[5] Fang Y, Ginsberg C, Sugatani T, Monier-Faugere MC, Malluche H, Hruska KA (2014). Early chronic kidney disease-mineral bone disorder stimulates vascular calcification. Kidney Int.

[6] Savoia C, Burger D, Nishigaki N, Montezano A, Touyz RM (2011). Angiotensin II and the vascular phenotype in hypertension. Expert Rev Mol Med.

[7] Armstrong ZB, Boughner DR, Drangova M, Rogers KA (2011). Angiotensin II type 1 receptor blocker inhibits arterial calcification in a pre-clinical model. Cardiovasc Res.

[8] Kukida M, Mogi M, Kan-No H (2019). AT2 receptor stimulation inhibits phosphate-induced vascular calcification. Kidney Int.

[9] Tokumoto M, Mizobuchi M, Finch JL, Nakamura H, Martin DR, Slatopolsky E (2009). Blockage of the renin-angiotensin system attenuates mortality but not vascular calcification in uremic rats: sevelamer carbonate prevents vascular calcification. Am J Nephrol.

[10] Herencia C, Rodriguez-Ortiz ME, Munoz-Castaneda JR (2015). Angiotensin II prevents calcification in vascular smooth muscle cells by enhancing magnesium influx. Eur J Clin Investig.

[11] Friedewald WT, Levy RI, Fredrickson DS (1972). Estimation of the concentration of low-density lipoprotein cholesterol in plasma, without use of the preparative ultracentrifuge. Clin Chem.

[12] Fujimoto S, Watanabe T, Sakamoto A, Yukawa K, Morimoto K (1968). Studies on the physical surface area of Japanese. 18. Calculation formulas in three stages over all ages. Nihon Eiseigaku Zasshi.

[13] Kinugasa M, Mori S, Takaya T (2016). Serum phosphate is an independent predictor of the total aortic calcification volume in non-hemodialysis patients undergoing cardiovascular surgery. J Cardiol.

[14] Lomashvili KA, Wang X, Wallin R, O'Neill WC (2011). Matrix Gla protein metabolism in vascular smooth muscle and role in uremic vascular calcification. J Biol Chem.

[15] Sheehan J, White A (1982). Diuretic-associated hypomagnesaemia. Br Med J (Clin Res Ed).

[16] Sakaguchi Y, Hamano T, Obi Y (2019). A randomized trial of magnesium oxide and Oral carbon adsorbent for coronary artery calcification in Predialysis CKD. J Am Soc Nephrol.

[17] Darabian S, Luo Y, Homat A (2015). CAC score as a possible criterion for administration of angiotensin converting enzyme inhibitors and/or angiotensin receptor blockers: the MultiEthnic study of atherosclerosis. Coron Artery Dis.

[18] Vattikuti R, Towler DA (2004). Osteogenic regulation of vascular calcification: an early perspective. Am J Physiol Endocrinol Metab.

[19] Shimizu H, Nakagami H, Osako MK (2008). Angiotensin II accelerates osteoporosis by activating osteoclasts. FASEB J.

[20] Kaneko K, Ito M, Fumoto T (2011). Physiological function of the angiotensin AT1a receptor in bone remodeling. J Bone Miner Res.

[21] Yamamoto S, Kido R, Onishi Y (2015). Use of renin-angiotensin system inhibitors is associated with reduction of fracture risk in hemodialysis patients. PLoS One.

