# Supplementary material for: User of angiotensin-converting-enzyme inhibitor and/or angiotensin II receptor blocker might be associated with vascular calcification in predialysis chronic kidney disease patients: a retrospective single-center observational study: ACEI/ARB and vascular calcification
Source: BMC Nephrol. 2021 Jan 6;22:7. doi: 10.1186/s12882-020-02198-6 (PMC7789142; doi:10.1186/s12882-020-02198-6)
Supplement: Supplementary file 1 — Additional file 1. [file 12882_2020_2198_MOESM1_ESM.docx]

**Supplementary Data**

Patients characteristics among Group1, Group 2 and Group 3

|  | **Group 1**  **(N=42)** | **Group 2**  **(N=7)** | **Group 3**  **(N=41)** | **P** |
| --- | --- | --- | --- | --- |
| Age (y) | 73 (61 – 79) | 72 (62 − 84) | 71 ±10 | 0.8848 |
| Sex (Male %) | 50 | 57 | 71 | 0.1540 |
| Alb (g/dL) | 2.82 ± 0.90 | 2.5 (2.0 − 3.0) | 3.1 (2.4 − 3.7) | 0.2084 |
| Corrected Ca (mg/dL) | 9.44 ±0.78 | 9.8 (9.0 − 10.3) | 9.4 ± 0.76 | 0.6658 |
| P (mg/dL) | 3.9 (3.2 – 4.2) | 4.4 (3.6 − 4.9) | 3.7 ± 1.1 | 0.2021 |
| Corrected Ca x P Product | 36.4 (32.1 – 41.8) | 41.2 (37.1−48.0) | 34.6 ± 9.2 | 0.1281 |
| log[eGFR] | 3.11 (2.62 – 4.18) | 2.91 (2.30 − 4.05) | 2.95 (2.38−3.65) | 0.2367 |
| P binder with Ca (%) | 2.4 | 14.3 | 4.9 | 0.3614 |
| P binder without Ca (%) | 4.8 | 28.6 | 7.32 | 0.0924 |
| DM (%) | 23.8 | 71.4 | 56.1 | 0.0032 |
| SBP (mmHg) | 130 (120 – 145) | 146 (127 − 148) | 133 ± 24 | 0.2832 |

Sex, P binder with Ca, P binder without Ca, and DM among Group 1, Group 2 and Group 3 were analyzed with Chi square test. The other parameters were analyzed with Kruskal-Wallis test.

Alb, Albumin; Ca, Calcium; P, Phosphate; eGFR, estimated glomerular filtration rate; DM, diabetes mellitus; SBP, systolic blood pressure
